# An Organic Flexible Artificial Bio-Synapses with Long-Term Plasticity for Neuromorphic Computing

**DOI:** 10.3390/mi9050239

**Published:** 2018-05-15

**Authors:** Tian-Yu Wang, Zhen-Yu He, Lin Chen, Hao Zhu, Qing-Qing Sun, Shi-Jin Ding, Peng Zhou, David Wei Zhang

**Affiliations:** State Key Laboratory of ASIC and System, School of Microelectronics, Fudan University, Shanghai 200433, China; wangtianyu16@fudan.edu.cn (T.-Y.W.); 17212020012@fudan.edu.cn (Z.-Y.H.); hao_zhu@fudan.edu.cn (H.Z.); qqsun@fudan.edu.cn (Q.-Q.S.); sjding@fudan.edu.cn (S.-J.D.); pengzhou@fudan.edu.cn (P.Z.); dwzhang@fudan.edu.cn (D.W.Z.)

**Keywords:** flexible organic electronics, artificial synapses, neuromorphic computing, long-term plasticity

## Abstract

Artificial synapses, with synaptic plasticity, are the key components of constructing the neuromorphic computing system and mimicking the bio-synaptic function. Traditional synaptic devices are based on silicon and inorganic materials, while organic electronics can open up new opportunities for flexible devices. Here, a flexible artificial synaptic device with an organic functional layer was proposed. The organic device showed good switching behaviors such as ON/OFF ratio over 100 at low operation voltages. The set and reset voltages were lower than 0.5 V and −0.25 V, respectively. The long-term plasticity, spike-timing-dependent plasticity learning rules (STDP), and forgetting function were emulated using the device. The retention times of the excitatory and inhibitory post-synaptic currents were both longer than 60 s. The long-term plasticity was repeatable without noticeable degradation after the application of five voltage pulse cycles to the top electrode. These results indicate that our organic flexible device has the potential to be applied in bio-inspired neuromorphic systems.

## 1. Introduction

The human brain can be seen as an effective system that is capable of analyzing complicated tasks through the integration of storage and computation [1,2]. To date, a neuromorphic computing system has been proposed and developed to overcome the bottleneck of classical von Neumann computers [3]. It has been widely recognized that fabricating an artificial electronic device with the function of mimicking the behaviors of a bio-synapse is necessary to realize neuromorphic computing. Many synaptic behaviors have been emulated using artificial synaptic devices, including long-term potentiation (LTP), long-term depression (LTD), paired-pulse facilitation (PPF), and STDP (spike-timing-dependent plasticity) [4]. The conductance of devices should be modulated gradually and simulate weight changes of bio-synapses [5,6], which are the fundamental to achieving synaptic plasticity. In recent years, various devices including CMOS transistors, resistive random access memory (RRAM), ferroelectric random access memory (FeRAM), and phase-change memory (PCM) have been demonstrated exhibiting such synaptic behaviors [4,6,7,8]. Among them, RRAM with the advantages of high-integration, low-power consumption, and simple structure, has become one of the promising candidates for the applications in neuromorphic computing. 

On the other hand, flexible electronics has attracted more interests of researchers and has been widely studied because of the potential in future wearable devices [9]. Flexible electronics are more portable and deformable in comparison with silicon-based devices [10,11,12]. However, most RRAM-based synaptic memories are composed of various inorganic materials, such as HfO_x_, Al_2_O_3_, and ZnO [13,14,15]. These inorganic materials usually require high temperature treatment steps with poor stretchability. In addition, the intrinsic properties of these materials are not compatible with a flexible substrate, which cannot meet the development and applications of flexible electronics [15,16,17]. Therefore, it is urgent to find a type of material suitable for flexible devices. There are many reports showing that organic materials can avoid high temperature treatment [18,19], which can be applied as the functional layers of flexible RRAM. Organic polymers have the advantages of simple preparation process at room temperature, low cost, and good stretchability. Poly(3,4-ethylenedioxythiophene): poly(styrenesulfonate) (PEDOT:PSS) is one of the common polymers with excellent stretchability [20,21], which has been proven to have resistive switching behaviors [22] and synaptic plasticity separately on the rigid substrate [23,24]. However, the realization of abrupt bipolar resistive switching characteristics and mimicking synaptic behaviors at the same time on a flexible substrate with PEDOT:PSS has not been reported.

Here, we fabricated a flexible RRAM based on PEDOT:PSS and examined its current response to different voltages. The device showed excellent resistive switching characteristics under direct-current sweep. It turned from high resistance state (HRS) to low resistance state (LRS) and came back to HRS at low operation voltages. Furthermore, good synaptic plasticity, including LTP, LTD, forgetting curve, and STDP were demonstrated under pulse chains in this flexible synaptic device. The controllable conductance is related to the transformation and migration of PEDOT^+^ [25]. These results demonstrate the feasibility of flexible PEDOT:PSS-based RRAM used as artificial synapses for neuromorphic computing and the potential for wearable electronics applications [26,27].

## 2. Materials and Methods

The preparation of active layer was processed with the solution of PEDOT:PSS (Clevios PH1000), which was purchased from Heraeus (Germany). Before coating, the PEDOT:PSS solution was filtered through a micro filter membrane with pore size of 0.22 μm.

**Figure 1:**
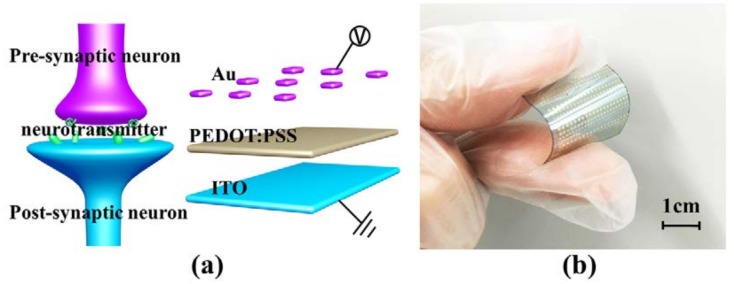
(**a**) The schematic structure of a bio-synapse and the corresponded PEDOT:PSS-based RRAM; (**b**) optical image of our flexible synaptic device in the bend state.

The synaptic device made by us has a structure of Indium Tin Oxides (ITO)/PEDOT:PSS/Au with a cross-sectional junction circle of 200 um diameter, as shown in Figure 1a. Polyethylene terephthalate (PET) was adopted as the flexible substrate. The substrate of ITO-coated PET was cleaned by mixing detergent of acetone and isopropyl alcohol (IPA) in an ultrasonic bath for 5 min. Then, the substrate was treated with oxygen plasma at 150 W for 3 min, and the film of PEDOT:PSS was spin-coated on the ITO electrode followed by baking at 120 °C for 10 min on a hotplate. The electrode of Au was deposited on PEDOT:PSS with a shadow mask by physical vapor deposition(PVD, Sputter system M362, SPECS), as shown in Figure 1b. 

All measurements of PEDOT:PSS-based RRAM were at room temperature, and atmospheric pressure biased the top electrode (Au) and grounded the bottom electrode (ITO). The electrical characteristics of the device were performed using Agilent B1500A and B1525 semiconductor parameter analyzer. The resistive switching characteristics were achieved by applying DC voltage with B1500A to the top electrode of the device. Two pulse channels of the B1525 were used to input pre- and post-synaptic pulses and induce synaptic behaviors of our device. The surface topography of PEDOT:PSS film was obtained by field emission scanning electron microscope (FESEM, ZEISS-SIGMA HD), as shown in Figure S1.

## 3. Results and Discussion

The two terminal structure of the flexible PEDOT:PSS-based RRAM is suitable for large-scale preparation of artificial synaptic arrays [28]. As shown in Figure 1a, the top electrode and bottom electrode corresponds to pre- and post-synaptic neuron, respectively. 

**Figure 2:**
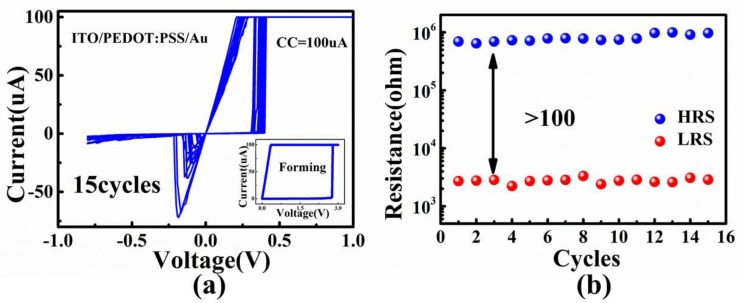
(**a**) The I-V curves of the flexible PEDOT:PSS-based RRAM. Inset shows the forming process of the device; (**b**) the HRS and LRS measured by DC sweeping of the device.

Figure 2 demonstrated the resistive switching characteristics of the flexible RRAM. The forming voltage was ~3 V, and the operation voltage was very low, i.e., the set voltage is lower than 0.5 V, and the reset voltage is lower than −0.25 V. A 0 V→1 V→0 V→−0.8 V→0 V DC voltage cycle was applied to the top electrode of Au with the ITO bottom electrode grounded. We realized the typical bipolar characteristics for the ITO/PEDOT:PSS/Au. The current-voltage (I–V) characteristics of our device through 15 cycles were measured. The device showed stable set operations under a compliance current (CC) of 100 uA in Figure 2a. The device showed high ON/OFF ratio larger than 100, as shown in Figure 2b. The resistive switching behaviors were linked to the regeneration and rupture of PEDOT^+^ conductive paths because of the injection and extraction of the hole in the film under the positive and negative voltages [29]. When the positive voltage was applied to the top electrode, the hole injected to PEDOT^0^, and it turned to PEDOT^+^. PEDOT^+^ was more conductive than PEDOT^0^ and could be accumulated to form a conductive path in the film.

**Figure 3:**
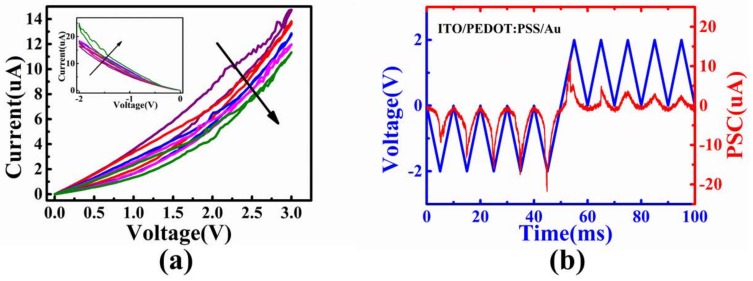
(**a**) I-V curves during five DC sweeps from 0 V to 3 V of PEDOT:PSS-based RRAM. Inset shows the I-V curves under five negative voltage sweeps from 0 V to −2 V; (**b**) the modulated currents (red lines) under 5 constructive negative pulse (−2 V, 10 ms) and positive pulse (2 V, 10 ms) trains (blue lines).

To examine the application of artificial synaptic device in synaptic plasticity, we applied continuous positive or negative voltages to the top electrode of Au. Both positive and negative voltages were carefully chosen to avoid abrupt resistance switching behaviors. As shown in Figure 3a, there were five consecutive sweeps of positive voltages or negative voltages. With the positive voltages swept from 0 V→3 V→0 V, the conductance decreased after each cycle (Figure 2a). In contrast, the conductance increased gradually under 5 negative DC sweeps (0 V→−2 V→0 V), which was similar as potential behaviors in bio-synapses. To clearly show the successful modulation of synaptic weights in our device, pulse training mode was utilized. The pulse amplitude was 2 V, and the pulse width was 10 ms without intervals, which was indicated by the blue lines in Figure 3b. The red lines were the current response according to 10 consecutive pulse training. The conductance of our device was potentiated by 5 negative bias pulses and depressed by 5 positive bias pulses, showing the potential for LTP and LTD under pulse tests.

**Figure 4:**
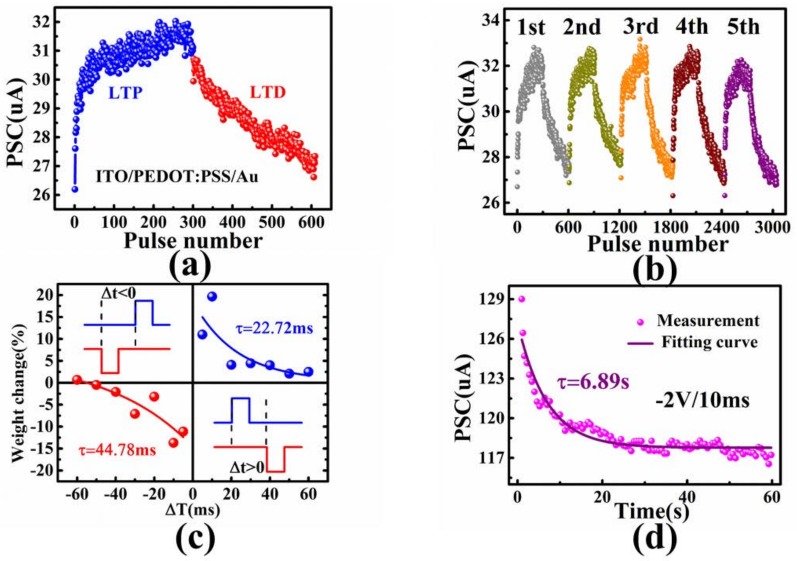
(**a**) The LTP and LTD under 300 negative pulses (−1.5 V, 10 ms) and 300 positive pulses (1 V, 10 ms) of our PEDOT:PSS-based RRAM; (**b**) Five operations of LTP and LTD; (**c**) simulation of STDP by changing the pulse intervals of pre- and post-synaptic spiking; (**d**) forgetting curve after a single pulse (−2 V,10 ms). The responded current was read at 0.1 V.

Gradual modulated conductance is important for a synaptic device towards neuromorphic computing [30]. We applied 300 negative pulses (−1.5 V, 10 ms) followed by 300 read operations with pulses of 0.1 V. The post-synaptic current under the read voltages increased gradually, which corresponded to the LTP behavior of bio-synapses. Similarly, 300 positive pulses (1 V, 10 ms) and read pulses of 0.1 V were applied to our devices, which successfully emulated the LTD behavior (Figure 4a). The response of current during measurement of LTD was shown in Figure S2, and the schematic of pulse waveform was shown in Figure S3. Furthermore, we repeated the pulse chains 5 times, and the device showed excellent endurance (Figure 4b). The device showed STDP learning rules in Figure 4c, which described the relationship between weight change (*ΔW*) and time interval (*Δt*). The time interval was defined as follows:

*Δt* = *t*_post_ − *t*_pre_, (1)

in which the *t*_post_ and *t*_pre_ were the time of pulse come to the post-synaptic and pre-synaptic electrode. A pair of pulses was applied to the pre-synaptic electrode (−1.5 V, 10 ms) and post-synaptic electrode (1.5 V, 10 ms). Additionally, the weight change was described as follows:

*ΔW* = (*G_t_* − *G*_0_)/*G*_0_ (2)

in which *G_t_* was the conductance of the device at the time node of “*t*” and *G*_0_ was the initial conductance of the device at the time node of “*t* = 0”. When the pulse was exerted earlier on the pre-synapse, *Δt* was greater than 0. The weights increased, which indicated that the connection relationship between two synapses was potentiated. In contrast, the weights decreased, which indicated that the connection strength of two synapses was weakened (*Δt* < 0).

Forgetting curve is a common bio-synaptic behavior, which has been studied widely and reported [2,11,31]. The relaxation of post-synaptic current after spiking pulse to the pre-synaptic electrode could be used for mimicking the forgetting function in psychology. Figure S4 showed the whole process during the measurement of the forgetting function of the device. As shown in Figure 4d, after applying one pulse (−2 V, 10 ms) to the pre-synaptic electrode of Au, currents of post-synaptic electrode were recorded at the read voltage of 0.1 V for 60 s. The relationship between post-synaptic current and time was fitted by an exponential decay equation as follows:

*I_t_* = *I*_0_ + *A* exp(*−t*/τ), (3)

in which *I_t_* and *I*_0_ were the memory current at time of *t* and stabilized state, *A* was the prefactor, and τ was the relaxation time constant, which illustrated the forgetting speed of the memory. The forgetting curve was fitted based on Equation (3), in which τ was 6.89 s.

**Figure 5:**
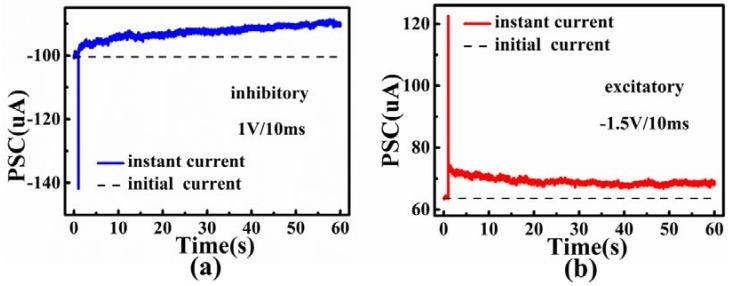
The retention behaviors of (**a**) inhibitory of post-synaptic currents under one pulse (1 V, 10 ms) and (**b**) excitatory features of post-synaptic currents under one pulse (−1.5 V,10 ms).

As a nonvolatile memory, retention characteristic is a significant indicator. We emulated the inhibitory and excitatory features of bio-synapses using post-synaptic current. Upon the application of positive (1 V, 10 ms) and negative (−1.5 V, 10 ms) pulses, the post-synaptic current could hold at least 60 s (Figure 5). Excitatory or inhibitory of PSC could modulate the synaptic connection synergistically. After a negative pulse (−1.5 V, 10 ms) was implemented at the top electrode, the PSC increased obviously, and the change of current between the resting current after 60 s and the instantly stimulated current was kept above 60%. After a positive pulse (1 V, 10 ms) was implemented at the top electrode, the PSC decreased obviously, and the change of current was kept above 100% after 60 s. The life time of the PSC was much longer than 60 s, indicating that LTP and LTD could be realized by multiple pulses with time intervals shorter than 60 s.

## 4. Conclusions

We fabricated a flexible PEDOT:PSS-based artificial synaptic device that exhibited not only abrupt resistive switching of binary characteristic but also gradual muti-level conductance modulation used for mimicking synaptic plasticity. The Au top electrode and ITO bottom electrode corresponded to the pre- and post-synapse. We applied voltage to the top electrode and recorded the responded current of the bottom electrode to assess the device characteristics. The device showed good resistive switching behaviors with the ON/OFF ratio larger than 100. The set and reset voltage was lower than 0.5 V and −0.25 V, respectively. Besides, LTP, LTD, STDP, and forgetting function were manifested in our device. After five repeatable tests with 300 positive and 300 negative pulses for LTD and LTP, there was no obvious degradation observed in the device. These results suggested that our two terminal organic flexible RRAMs had the potential for neuromorphic computing.

## Supplementary Materials

The following are available online at http://www.mdpi.com/2072-666X/9/5/239/s1: Figure S1: SEM image of PEDOT:PSS film; Figure S2: transient response during measurement of LTD; Figure S3: continuous pulses used for measuring the LTD; and Figure S4: the response of currents during measuring the forgetting curve.
