# Supplementary material for: An Organic Flexible Artificial Bio-Synapses with Long-Term Plasticity for Neuromorphic Computing
Source: Micromachines (Basel). 2018 May 15;9(5):239. doi: 10.3390/mi9050239 (PMC6187857; doi:10.3390/mi9050239)
Supplement: Supplementary file 1 [file micromachines-09-00239-s001.pdf]

Supporting Information

An Organic Flexible Artificial Bio-synapses with Long-Term Plasticity For Neuromorphic Computing

Tianyu Wang[1], Zhenyu He[1], Lin Chen[1*], Hao Zhu[1], Qing-Qing Sun[1], Shi-Jin Ding[1], Peng Zhou[1], and David Wei Zhang[1]

[1]*State Key Laboratory of ASIC and System, School of Microelectronics, Fudan University, Shanghai 200433.*

Keywords: flexible organic electronics; artificial synapses; neuromorphic computing; long-term plasticity

* Corresponding author: linchen@fudan.edu.cn, Room 403, Microelectronics Building, Fudan University, No. 220, Handan Road, Shanghai, 200433

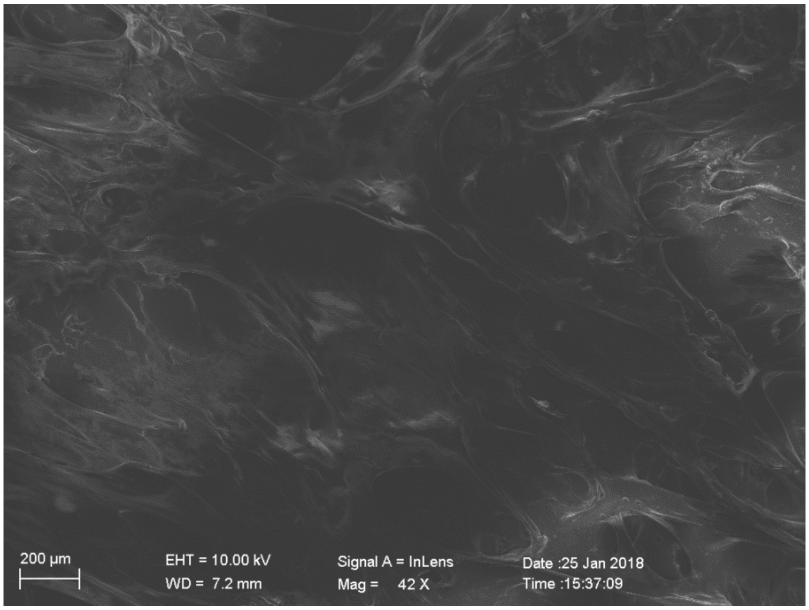

Figure S1: SEM image of PEDOT:PSS film. The PEDOT:PSS film was coated on the silicon to obtain its surface topography using SEM. During the testing process, the extremely high electrical voltage was set as 10kV.

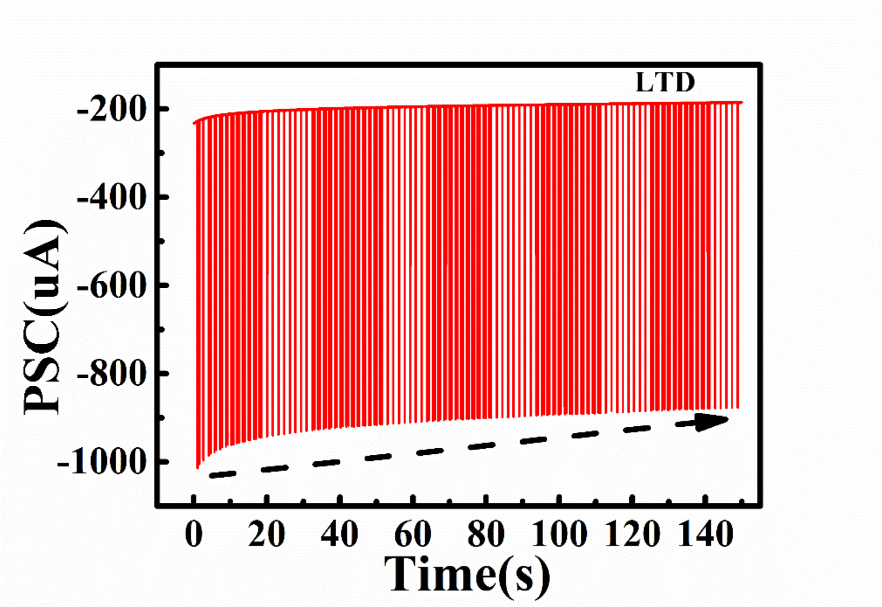

Figure S2: Transient response during measurement of LTD. With continuous pulse trains, the device show gradual change of transient post-synaptic currents. The current decreased with the increase of pulse number. The arrow represent the trend of post-synaptic current with applied pulses. As the voltage was applied to the top electrode continuously, the current of post synapse gradually increased.

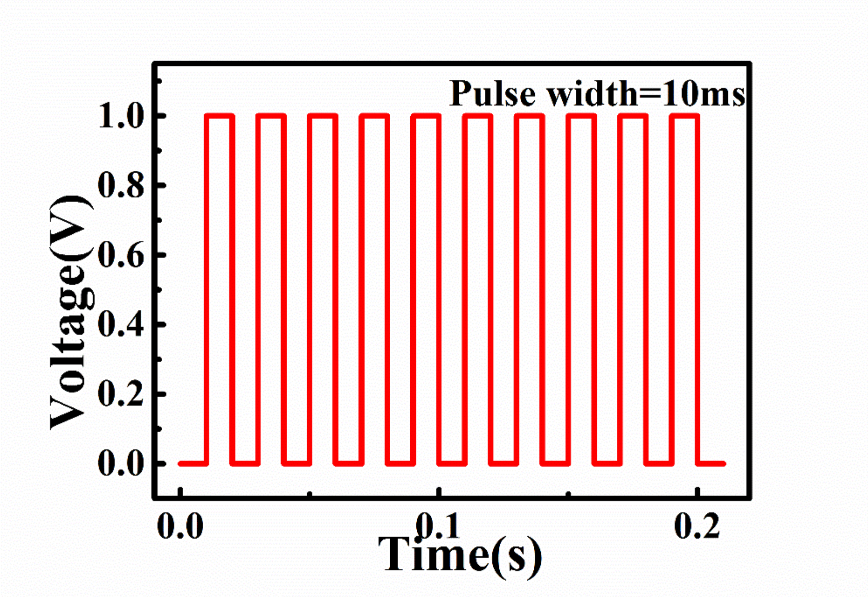

Figure S3: Continuous pulses used for measuring the LTD. There were 10 continuous pulses with the pulse width of 10ms and pulse amplitude of 1V. During measurement of LTD, there were 300 continuous pulses as shown in Figure S3.

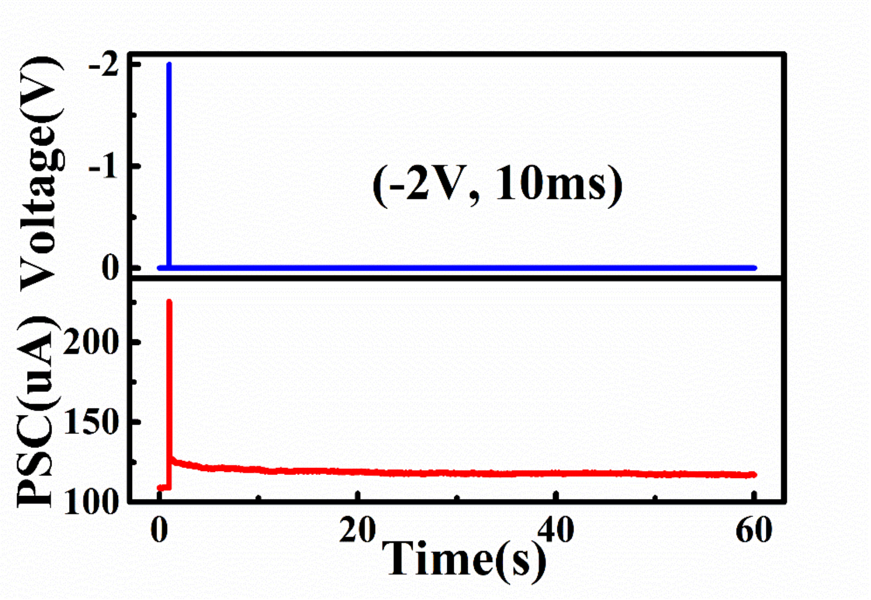

Figure S4: The response of currents during measuring the forgetting curve. The pre-synaptic electrode was applied a pulse with the pulse width of 10ms and pulse amplitude of -2V. The whole process during measurement was recorded under the read voltage of 0.1V.
